# Estimating ‘Lost Heart Beats’ Rather than Reductions in Heart Rate during the Intubation of Critically-Ill Children

**DOI:** 10.1371/journal.pone.0086766

**Published:** 2014-02-04

**Authors:** Peter Jones, Nick Ovenden, Stéphane Dauger, Mark J. Peters

**Affiliations:** 1 Critical Care Group – Portex Unit, Institute of Child Health, University College London, London, United Kingdom; 2 Paediatric Intensive Care – Réanimation Pédiatrique, Assistance Publique-Hôpitaux de Paris, Hôpital Robert Debré, Paris, France; 3 Department of Mathematics, University College London, London, United Kingdom; 4 Université Paris Diderot, Sorbonne Paris Cité, Paris, France; Rutgers University, United States of America

## Abstract

**Purpose:**

Reductions in heart rate occur frequently in children during critical care intubation and are currently considered the gold standard for haemodynamic instability. Our objective was to estimate loss of heart beats during intubation and compare this to reduction in heart rate alone whilst testing the impact of atropine pre-medication.

**Methods:**

Data were extracted from a prospective 2-year cohort study of intubation ECGs from critically ill children in PICU/Paediatric Transport. A three step algorithm was established to exclude variation in pre-intubation heart rate (using a 95%CI limit derived from pre-intubation heart rate variation of the children included), measure the heart rate over time and finally the estimate the numbers of lost beats.

**Results:**

333 intubations in children were eligible for inclusion of which 245 were available for analysis (74%). Intubations where the fall in heart rate was less than 50 bpm were accompanied almost exclusively by less than 25 lost beats (n = 175, median 0 [0–1]). When there was a reduction of >50 bpm there was a poor correlation with numbers of lost beats (n = 70, median 42 [15–83]). During intubation the median number of lost beats was 8 [1]–[32] when atropine was not used compared to 0 [0–0] when atropine was used (p<0.001).

**Conclusions:**

A reduction in heart rate during intubation of <50 bpm reliably predicted a minimal loss of beats. When the reduction in heart rate was >50 bpm the heart rate was poorly predictive of lost beats. A study looking at the relationship between lost beats and cardiac output needs to be performed. Atropine reduces both fall in heart rate and loss of beats. Similar area-under-the-curve methodology may be useful for estimating risk when biological parameters deviate outside normal range.

## Introduction

There is a risk of haemodynamic instability leading to death during intubation during critical illness (CCI). The action and interaction of two different mechanisms are responsible for such instability; the pre-intubation disruption of cardiovascular reserve and/or the influence of physiologic and pharmacologic disturbances during intubation. Adult studies report mortality between 1–3% and have focussed on pre-intubation risk factors such as hypotension or the need for inotropes [1]–[3]. Mortality in children is lower at 0.4% [4], [5]. Our recent study in children has demonstrated that the relatively frequent reductions in heart rate are unrelated to death during intubation [4].

Physiologic reductions in heart rate may occur due to the reflex activation of the Vagus nerve by mechanical stimulation of the laryngopharynx and/or hypoxia [6], [7]. Alternatively, they may occur due to the action of induction agents and/or depolarising neuromuscular blockers [8]–[10]. The choice of measuring heart rate changes during CCI in children is due to the relative parasympathetic predominance in early life which increases the likelihood of vagal activation [11].

When describing changes in heart rate during paediatric intubation, some studies look at the lowest heart rate observed, [12], [13] or the percentage reduction from baseline [14]. Other studies describe the proportion of cases below a normal-for-age threshold for healthy children at rest [15]–[17], or use a subjective definition of *‘clinically significant bradycardia’* [18]. The numerical data are superior in describing the variation in heart rate, whereas categorical data attempt to differentiate normal from abnormal. Unfortunately, neither approach considers the variation in pre-intubation heart rate or the duration of reduction in heart rate.

Our objective was to establish an algorithm to estimate the loss of heart beats during intubation that would eliminate pre-intubation heart rate variation whilst taking into account both the reduction in heart rate and the time of reduction in heart rate and compare this to reduction in heart rate alone. We also tested this approach by describing the impact of atropine pre-medication.

## Methods

This prospective, observational, cohort study was conducted between September 2007 and September 2009 at the Hôpital Robert Debré, Paris, France. Ethical permission for the study was approved by the Comité d'Ethique et d'Evaluation en Recherche Biomédicale of the Groupe Hospitalo-Universitaire-Paris Nord. An information letter was provided for parents detailing the nature of the study, the receipt of which was not documents in patients' notes. This procedure is in accordance with French law.

All children who were undergoing their first intubation, who were not in asystole, and were aged less than eight years, were eligible for inclusion. ECG recordings during intubation were made in the Paediatric Intensive Care Unit (PICU) and by the Paediatric/Neonatal Intensive Care Transport Service (ICT).

The first step of the algorithm involved the elimination of pre-intubation heart rate variation by the definition of a limit below which beats were lost. A one-minute recording of pre-intubation baseline heart rate was made (ECG strip 25 mm/s). Two Paediatric Intensivists independently estimated the lowest and highest heart rate from the longest and shortest two consecutive R-R intervals. The lowest heart rate was subtracted from the highest heart rate, to give a value for the variation in baseline heart rate. The standard deviation of the variation in baseline heart rate for the included children was calculated, divided by two (as above and below the mean) and multiplied by 1.96 to give a 95% confidence interval (*C*). When subtracted from the mean pre-intubation heat rate of any individual this demarcates the limit below which beats could be lost.

The second step involved the reading of the continuous ECG recordings which started from the moment of insertion of the laryngoscope until the positioning of the endo-tracheal tube in the trachea and a SpO2 of >95%, or connection to a ventilator. Continuous heart rates during intubation were determined by the measurement of two consecutive R-R intervals every 5.5 seconds (d*t*) during any episode where the heart rate fell below the previously defined limit. An interval of 5.5 seconds was used because there was a regular break in the PICU ECG recordings between 7 and 11 seconds. So as not to record an almost infinite number of lost beats, the calculation of lost beats of any child who became asystolic was stopped at the moment of the last ECG complex. Point data in the ECG recordings were averaged between the previous and following 5.5 second interval for no more than one missing 5.5 second interval. Where data were missing for two consecutive intervals of 5.5 seconds the intubation was excluded. Those ECGs where more than 10% of data were missing were also excluded.

**Figure 1:**
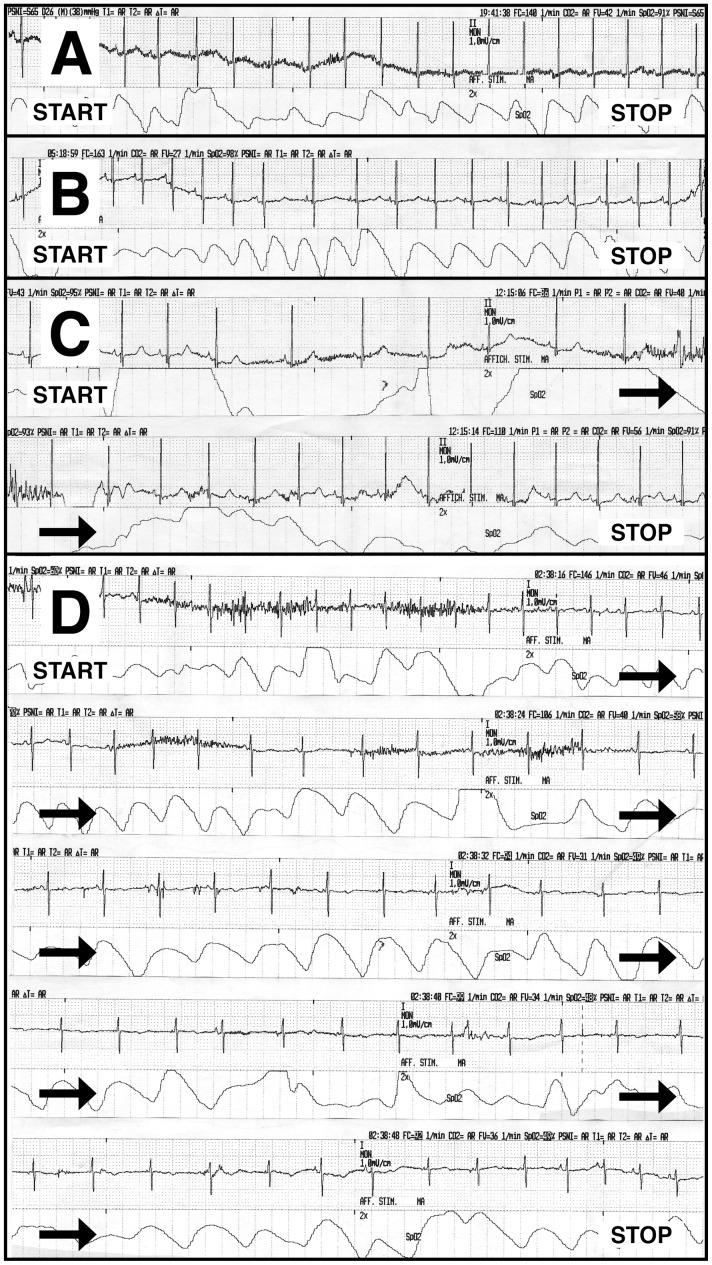
The ECGs of four intubations are shown to demonstrate loss of beats. Strip ‘A’ shows heart rate variability from a minimum of 135 beats/min to a maximum of 165 beats/min which is similar to that of the one minute pre-intubation strip, no loss of beats is recorded. Strip ‘B’ shows variation form 175 beats/min to a short deceleration of 108 beats/min which results in the loss of one heart beat. Strip ‘C’ shows a longer deceleration from 110 to 67 beats/min which results in the loss of six beats and strip ‘D’ a prolonged deceleration from 142 to 87 beats/min which results in the loss of 25 beats.

The third step involved the estimation of the number of lost beats according to the following formula; a total number of readings *N* (meaning that the total time interval measured was *T* =  (*N* −1) d*t* seconds) and a series of ECG values *e_i_*  =  *e*(*t_i_*) at each time interval *i* = 1,…*N* gave the total area below the lower 95% confidence interval of the heart rate variation prior to intubation. The integral represents the number of 'lost heart beats' (*LB*) by,
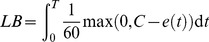



…where *t* and *T* are measured in seconds. A numerical trapezoidal approximation is used to calculate this interval. Figure 1 shows an illustration of four ECG recordings demonstrating the difference between heart rate variation without loss of beats and decelerations resulting in loss of beats.

The following data were also prospectively recorded: age, sex, pathology (neonatal respiratory distress [NRD], non-neonatal respiratory distress [non-NRD], cardiac, neurological, ear nose and throat [ENT], sepsis and ‘other’) and principal sedation drug. The prescription of all drugs including atropine was at the discretion of the attending Intensivist.

### Statistical analysis

Qualitative variables are described as numbers and percentages (%) and quantitative variables as median [quartiles] or mean (standard deviation) according to their Gaussian distribution. Independent t-tests or a Wilcoxon test were used for continuous data and a Chi^2^ test for categorical data. All statistical tests were 2-sided and the probability of a type 1 error (α) was determined at <0.05. All statistical tests were carried out using SPSS (version 19).

## Results

### Population characteristics

**Figure 2:**
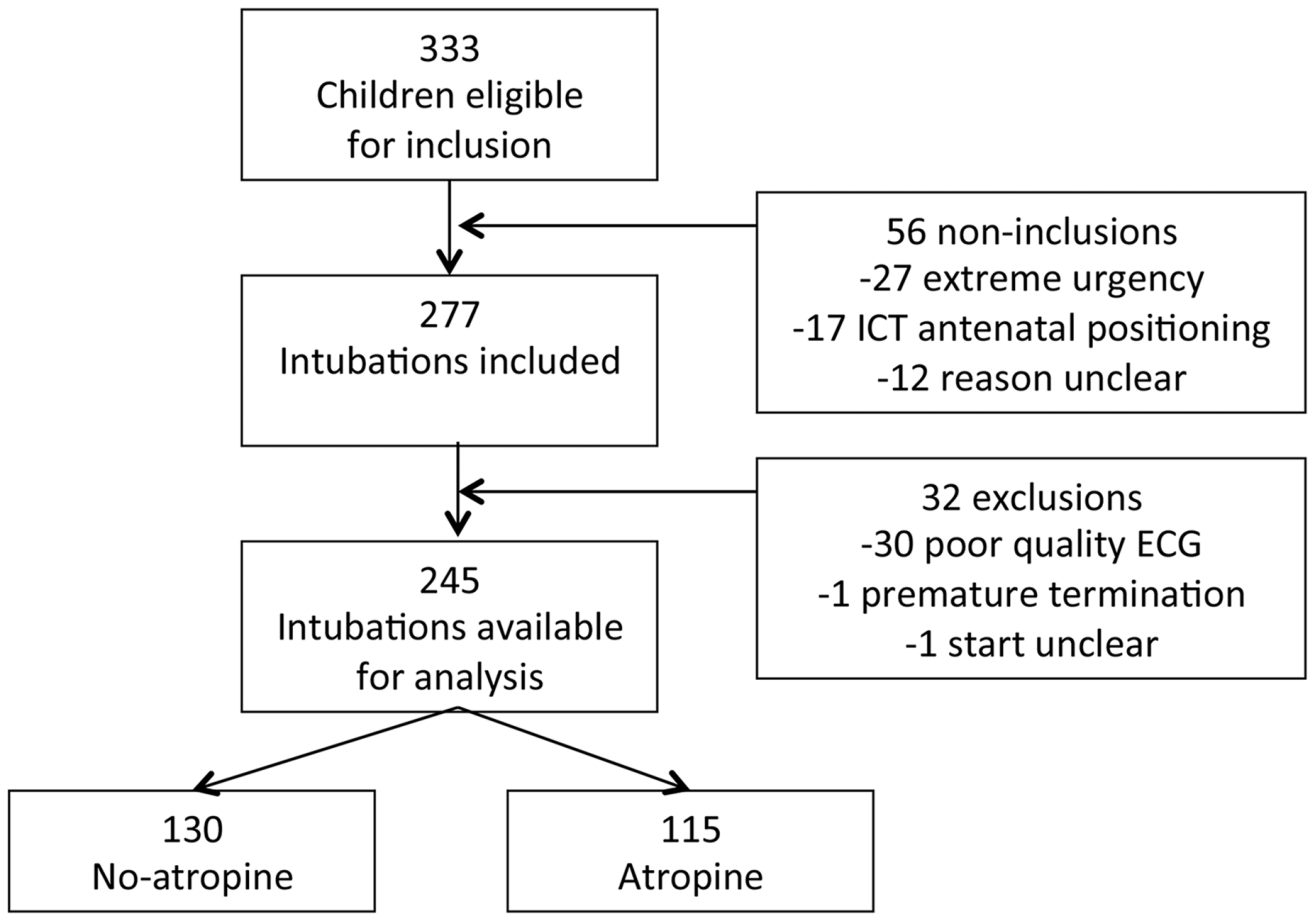
Flow chart of inclusions, non-inclusions and exclusions with the number of intubations available for analysis according to atropine use.

A total of 333 first intubations in children were eligible for inclusion, 277 intubations were included of which 245 were available for analysis (74%), see Figure 2 for the details for non-inclusions and exclusions. Ninety-eight children were included from PICU and 147 from ICT. Atropine was prescribed for 115 (47%) of intubations. The details of the baseline characteristics of the children are presented in Table 1. One child died during intubation with a lethal chromosomal disorder, pulmonary hypertension and septic shock.

**Table 1:** Population characteristics of the 245 children included.

	245 first intubations		
	No-atropine n = 130 (%)	Atropine n = 115 (%)	p
Age [days, IQR]	19 [0–188]	1 [0–67]	0.047*
Mean baseline heart rate, (beats/min, SD)	156 (123–179)	150 (124–176)	0.03*
Median variation in baseline heart rate [beats/min, IQR]	9 [5]–[15]	10 [6]–[16]	0.40
Sex (boys)	72 (55)	78 (68)	0.06
Neonatal respiratory distress	55 (42)	59 (51)	0.20
Non-Neonatal respiratory distress	36 (28)	16 (14)	0.01*
Cardiac	9 (7)	4 (3)	0.27
Ear nose and throat	8 (6)	8 (7)	0.80
Neurologic	10 (8)	18 (16)	0.07
Sepsis	9 (7)	7 (6)	1.00
Other	3 (2)	3 (3)	1.00
No drugs	34 (26)	35 (30)	0.48
Ethomidate	5 (4)	5 (4)	1.00
Propofol	38 (29)	20 (17)	0.04*
Ketamine	10 (8)	1 (1)	0.01*
Sufentanyl	14 (11)	19 (17)	0.20
Morphine	17 (13)	12 (10)	0.56
Midazolam	11 (8)	20 (17)	0.06
Other	1 (1)	3 (2)	0.63
Suxamethonium	4 (3)	6 (5)	0.52
Vecuronium	4 (3)	0	0.13

* Signifies a statistically significant difference, p<0.05.

### Comparison between reduction in heart rate and lost beats

The standard deviation of the variation in individual baseline heart rate was 14.5 beats-per-minute (bpm), which when multiplied by 1.96 gave the 95%CI. Half of the 95%CI (14.2 bpm) was subtracted from the mean for any individual patient to determine the limit of pre-intubation variability beyond which beats were lost.

**Figure 3:**
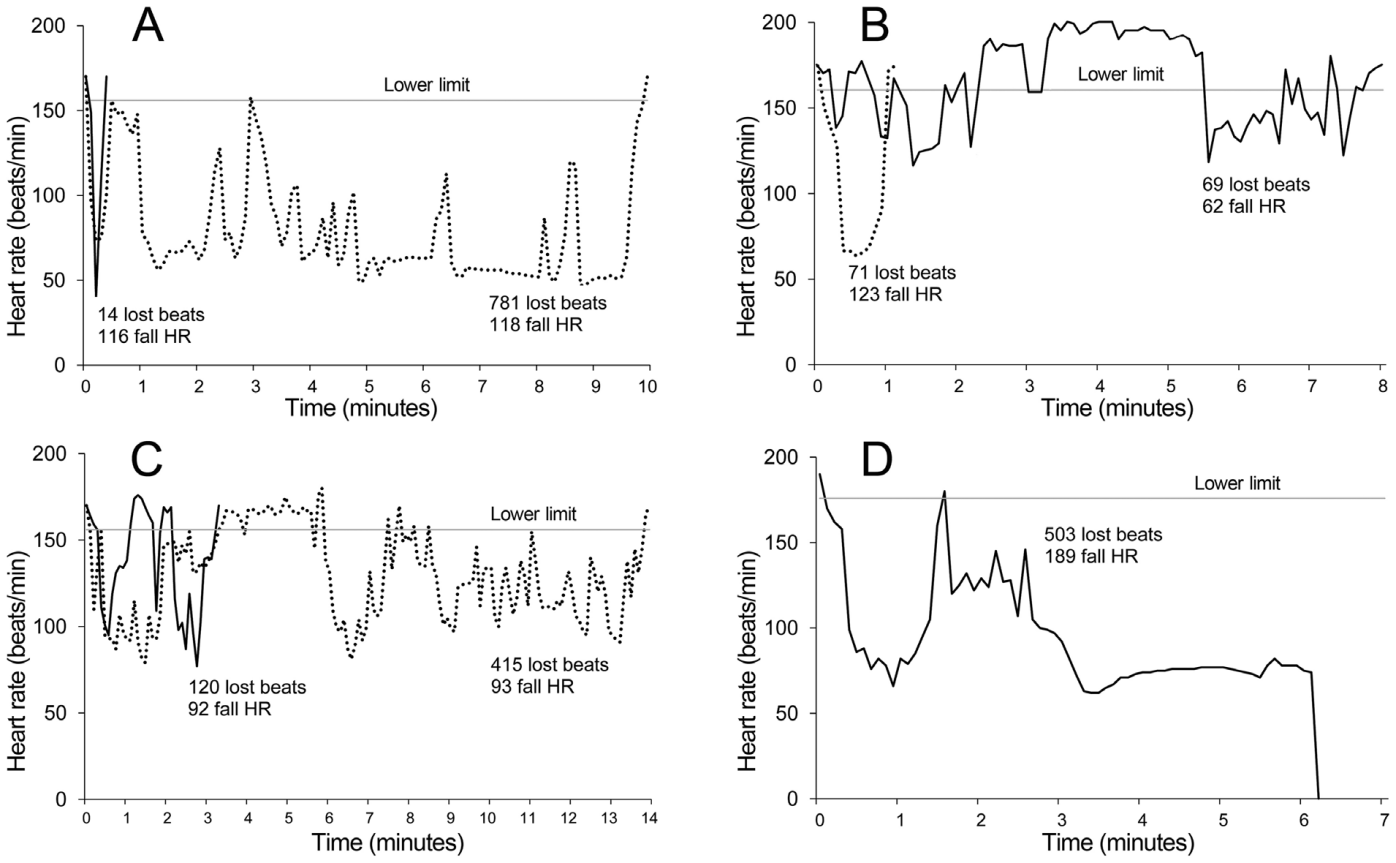
Changes in heart rate during several different intubations. Two intubations (dotted and solid lines representing different intubations from separate children) are shown for comparison of how the loss of heart beats can be poorly correlated with reduction in heart rate. A, shows two intubations with similar fall in heart rate (fall HR) and more than 50 fold differences in lost beats, B, shows two intubations where the number of lost beats was similar but the fall in heart rate 50% greater, C, shows two examples where there was a more than a three-fold difference in lost beats against reduction in heart rate and D, is the one child who died in the study having lost 503 beats before an asystolic arrest. Inter-bradycardia tachycardia is also apparent in B. Note that the scales on the *x*-axes are different for each graph.

Several examples of intubations are illustrated in Figure 3. Similar falls in maximum heart rate could produce very different numbers of lost beats. The heart rate tracing of the child who died during intubation is illustrated in Figure 3D; 512 beats were lost prior to asystole.

**Figure 4:**
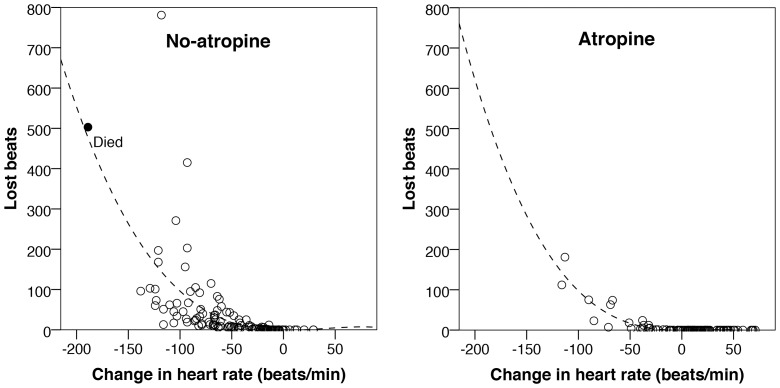
Distribution of the lost beats with and without atropine (n = 130 for no-atropine and n = 115 for with atropine). The r^2^ for no-atropine is 0.41 and 0.85 with atropine. Note the similarity between the two best-fit curves (dotted lines) both in terms of take off and position. All children survived intubation except that labelled ‘Died’.

All intubations where the fall in heart rate was less than 50 bpm were accompanied by less than 25 lost beats (n = 175, median 0 [0–1]) with the exception of one intubation where a fall in heart rate of 48 bpm registered 35 lost beats. When there was a reduction of >50 bpm there was a poor correlation with numbers of lost beats (n = 70, median 42 [15–83]), see Figure 4.

### Influence of atropine

Mean heart rate prior to intubation was 153 bpm (129–178). If no atropine was used during intubation, the mean heart rate and fell to 107 bpm (68–146) whereas it rose to a mean of 156pm (123–188) during intubation when atropine was used (p<0.001, mean difference 49 bpm, 95% CI 39–58). During intubation the median number of lost beats was 8 [1]–[32] when atropine was not used compared to 0[0–0] when atropine was used (p<0.001). When no atropine was used, 76% (99/130) of intubations involved lost beats whereas 19% (22/115) involved lost beats when atropine was used (p<0.001).

## Discussion

The lost beat score demonstrated that there was good correlation between reduction in heart rate and lost beats when the heart rate fell by no more than 50 bpm. Beyond a fall of 50 bpm there was poor correlation with the numbers of lost beats. Atropine pre-medication was associated with a significantly reduced number of lost beats and reduced heart rate.

### Linear no-threshold, non-linear no-threshold and area-under-curve models

Biological risk can be described as a function of intensity of exposure over time. For instance, underwater divers who experience short dives at deep depths can be of similar risk of decompression sickness as longer dives at shallower depth [19]. Similarly, ionizing radiation exposure is measured by intensity and as a factor of time [20]. Both of these risks are characterised by linear no-threshold (LNT) models whereby any increase in exposure equates to an increase in risk. However, in medicine LNT models are not generally used to estimate risk. Rather, risk is identified by deviation beyond normal ranges which are defined by the calculation of 95% confidence intervals using a cohort of normal individuals. For example, the concept of 95%CI (non-LNT) ranges for biochemical and haematological values is uniformly accepted. However, during illness, these normal ranges may no longer apply. One example is the mean heart rate for children adjusted to age [21] which is no longer representative in children with fever [22], [23].

The construction a non-LNT model using variance in pre-intubation heart rate for the first step of our algorithm is similar to methodology used to describe haematological and biochemical ranges. Thereafter we used an area-under-curve (AUC) model to integrate loss of beats from reduction in heart rate and length of time below the 95% threshold [24]. A trapezoidal function was used to approximate the point data and simplify calculation of the integral. The incorporation of a 95%CI threshold to is similar to an AUC model *with respect to increase*. Examples of AUC modelling are the calculation of risk of gentamycin toxicity during once daily dosing rather than measuring peak and trough levels [25] and measuring cortisol as a physiologic indicator of the responsiveness of the hypothalamic-pituitary-adrenal axis [26].

### Predicting haemodynamic instability

There are several possible advantages to our approach. Firstly, the use of a non-LNT threshold eliminates the measurement of pre-intubation variation in heart rate. Secondly, the model demonstrated that there is correlation between lost beats and moderate reduction in heart rate (<50 bpm). During intubation a reduction in heart rate of <50 bpm is relatively frequent and is compensated for by the simultaneous modulation of ejection volume and vascular resistance which maintains circulatory integrity. In addition, the relationship between lost beats and severe reduction in heart rate (>50 bpm) is poorly correlated. This reflects the clinical situation where a reduction in heart rate of >50 bpm is relatively infrequent but is not generally associated with haemodynamic instability. However, when haemodynamic instability occurs, it will almost inevitably include a reduction in heart rate of more than 50 bpm. Thirdly, using lost beats may better differentiate risk by highlighting intubations where there was a prolonged reduction in heart rate rather than simply a prolonged time from start to end of intubation.

Ideally the cardiac output (CO), which is a measure of whole body blood flow, should be used to estimate haemodynamic instability during intubation. A prolonged or substantial decrease in CO incurs risk of haemodynamic decompensation. The monitoring of heart rate gives a poor indication of risk of haemodynamic instability during intubation because CO is poorly correlated with heart rate in healthy, [27] premature, [28] and shocked [29] neonates. Currently the most precise indicator, and perhaps least controversial, indicator of haemodynamic decompensation is death during intubation. However, in this is a rare event (0.4%) in children [4], [5]. The limitation of our methodology is that the relationship between loss of beats and cardiac output has yet to be established. Such a study will probably need to be done in an animal experimental model. The concept of lost beats being detrimental to outcome is new. A correlation will be required to be made between a ‘gold standard’ outcome measure for haemodynamic instability for the hypothesis to be proven to be of practical value.

When the loss of beats was compared to reduction in heart rate with and without atropine there was no shift in the best-fit curve (Figure 4). However there was a change in the distribution the positioning of the intubations with a shift to the right when atropine was used. This may suggest that atropine may reduce, but not eliminate, risk of haemodynamic instability. Although there was good case-mix similarity between the atropine and non-atropine groups, this interpretation must be treated with caution due to the absence of randomisation or adjustment for bias in the study groups with and without atropine.

### Future model development

Monitoring equipment could be modified could present in real-time the number of beats lost to Intensivists which may facilitate the estimation of risk of circulatory failure. The use of similar methodology may benefit other situations where it is necessary to evaluate risk when normal ranges no longer apply. Examples could include the safety of permissive hypercapnia in extremely premature infants [30], hyperammonaemia from inborn errors of metabolism and neurological damage [31], and levels of haemoglobin and indication for transfusion in ventilated patients [32].

## Conclusions

A reduction in heart rate during intubation of <50 bpm reliably predicted a minimal loss of beats. When the reduction in heart rate was >50 bpm the heart rate was poorly predictive of lost beats and may be predictive haemodynamic instability. A study looking at the relationship between lost beats and cardiac output, which is the most reliable indicator of haemodynamic instability, needs to be performed. Atropine reduces both fall in heart rate and loss of beats. Similar area-under-the-curve methodology may be useful for measuring risk when biological parameters deviate outside normal range.
